# The Professor and the Quack

**Published:** 1885-02-20

**Authors:** 


### THE PROFESSOR AND THE QUACK.

BY A GEORGIA PHYSICIAN.

Whatever may be said in derogation of Homœpathy as a system, or of the absurdity of infinitesimal doses, it is conceded that, in the treatment of febrile affections of children, the homœpathists, as a rule, are remarkably successful.

Many years ago, during an unusual prevalence of infantile remittent and gastric fevers in Atlanta, I was struck with the superior success of a practitioner of this persuasion in that particular class of cases. It so happened, on one occasion, that I was called to a patient in the same building in which this my competitor in the healing art was also treating a case. Mine was a case of fracture of the femur, requiring much skill and care in its management. Both patients were children of the same parents, and this discrimination as to physicians in the two cases would seem to indicate the exercise of a better judgment on the part of our patrons, in some instances, than we are inclined to grant them; as to treat a fracture of an important part was something that required anatomical knowledge, tact, and skill, and not to be trusted to his homœpathic family physician, however great was his confidence in him in ordinary cases of sickness.

Being thus thrown into the presence of a patient who was being treated for remittent fever by a competitor whose success was the subject of frequent remark, I determined to ascertain, if possible, his plan of treatment. By a fortunate occurrence I discovered that his remedy was a solution of the *Tincture of aconite*, *radix*, five drops in a half glass of water, of which a teaspoonful was given every thirty minutes during the exascerbation, and a less quantity, and at longer intervals, during the remission. This, with a spare diet, cool applications to the head, and water to drink *ad libitum*, was the only treatment used. I noticed that the case, though a severe one, rapidly recovered.

Acting upon the instruction given by a distinguished, but somewhat eccentric, professor in one of our medical schools, long years ago, I took note of the fact, and have profited by it ever since, having used this same treatment for twenty years with far better results than I have ever derived from any other plan in such cases.

I am aware that aconite, in small and oft repeated doses, is now used by many physicians of all classes ; yet, at that time, it was rarely so used, but, when given, was in the large and hazardous doses of the old U. S. Dispensitory, of from five to twenty drops of the tincture.

The old professor above referred to advised his students to be close observers, and not to be above catching an idea, even though it might come from an old woman, or from a quack of any class or school. He was in the habit of telling to the class the following joke on himself :

On one occasion he had a troublesome and extremely obstinate case of what was regarded a neuralgic affection of the liver. For nearly two months he visited the patient daily, at a distance of ten miles in the country, the case resisting every remedy, and the patient, at last, reduced to what appeared to be the verge of death. On his last trip, fearing that he would find his patient dead, he inquired of every one he met if he had heard how Major Thompson was ? It was not until he had gotten within a mile of the residence of the patient that any information was obtained. Here, as he was crossing a creek, he met a rather rough-looking man, on a scrub and bony horse, and a saddle that, judging from its appearance, might have descended from his great-grandfather of the revolutionary war, and across which was a pair of pill-bags of enormous capacity. As our professor had no pill-bags or other insignia of his calling, the pill man did not suspect that he was a brother doctor. As their horses were drinking, the following colloquy ensued :

Professor—Do you reside in this neighborhood, sir ?

Pill-bags—Yas, sir ; not fur from here.

Professor—Have you heard how Major Thompson is to-day ?

Pill-bags—Yas, sir ; he is a great deal better; he is up and a walking about the house.

Professor—Is it possible ? It is reported that he is at the point of death.

Pill-bags—So it is, and so it was until they sent for me, which they did last night about 12 o'clock, supposin' him to be dyin' ; but it didn't take me no time to lift him out'n that bed !

Professor (very much astonished)—You astonish me, my dear sir. Will you be so kind as to tell me what you did to relieve him?

Pill-bags—Sartin'ly, stranger ; it was partly luck and partly science, fur I'm some doctor, ef I do say it myself. Now, the case is jest this : There has been a big bug from the city—one Professer C.—'tendin' on him, and goin' thar these eight or nine weeks, and all he done for him was no better than porein' water in a snake hole ! So, when I got thar, I jest 'quired of the family all about what the old professor had ben doin', detarmined to do somethin' else, come what mout ; and, from what I could gether, he done everything on God Almighty's arth, 'cept he hadn't give him a puke ; so, stranger, I hauled out my lobely and give him a hell of a puke, and dod drat my hide ef it didn't cure him quicker'n anything I ever seed, and no mistake, and I'm the man that done it !

The old professor used to tell the joke on himself at every session, and was, I fear, not a Christian man, as he always closed the joke with the following remark :

" Gentlemen, keep your eyes open, and don't be afraid of lowering your dignity by catching an idea, even if it comes from the lowest slums of empericism and quackery ; and remember, if you ever have a case of this kind, and don't know what else to do, *be sure to give him a hell of a puke !* "

